# Association between alcoholic beverage consumption and cerebral small vessel disease burden

**DOI:** 10.1016/j.tjpad.2025.100322

**Published:** 2025-08-05

**Authors:** Ben-Bo Xiong, Zi-Jie Wang, Zhi-Ming Li, Tian-Nan Yang, Xiang-Yu Li, Meng-Jie Lu, Qi Li

**Affiliations:** a Department of Neurology, The Second Affiliated Hospital of Anhui Medical University, No. 678 Furong Road, Shushan District, Hefei, China; b Department of Clinical Medicine, The Second Clinical Medical College, Anhui Medical University, Hefei, China; c Health Science Center, Ningbo University, Ningbo, China; d Department of Neurology, The First Affiliated Hospital of Chongqing Medical University, No.1 Youyi Road, Yuzhong District, Chongqing, China

**Keywords:** Alcohol consumption, Cerebral small vessel disease, White matter hyperintensities, Drinking frequency, UK biobank

## Abstract

**Background:**

The relationship between alcohol consumption and cerebral small vessel disease (CSVD) remains uncertain, particularly regarding drinking patterns and beverage types. We investigated how total alcohol intake, drinking frequency, and beverage-specific consumption are associated with CSVD burden using cross-sectional data.

**Methods:**

We included 27,326 UK Biobank (UKB) participants with MRI data, among whom 21,130 were current drinkers with full alcohol intake data. Alcohol consumption (frequency and beverage type) was self-reported. CSVD burden was measured via normalized white matter hyperintensity volume (WMHV) on T2-FLAIR MRI. Multivariable linear regression models adjusted for demographics, lifestyle, and vascular risk factors were used to examine associations.

**Results:**

Compared with non-drinkers, alcohol consumers had greater CSVD burden (Beta = 0.07; 95 % CI, 0.00–0.15). Among them, higher drinking frequency (≥5 times/week) was associated with increased CSVD burden (Beta = 0.10; 95 % CI, 0.07–0.13). High consumption of red wine, white wine/champagne, and spirits (≥7 servings/week) correlated positively with CSVD burden. In contrast, low-to-moderate beer/cider intake (≤3 servings/week) was inversely associated with burden. A dose-response relationship between total ethanol intake and CSVD burden was observed, with minimal intake (<1.97 g/day) showing a mild negative association, and higher levels increasing risk.

**Conclusion:**

Greater frequency and volume of alcohol intake, especially from wine and spirits, are linked with higher CSVD burden. Conversely, low beer/cider consumption may be inversely associated with CSVD burden. These findings underscore the importance of moderating alcohol consumption to maintain cerebrovascular health.

## Graphical abstract

**Figure:**
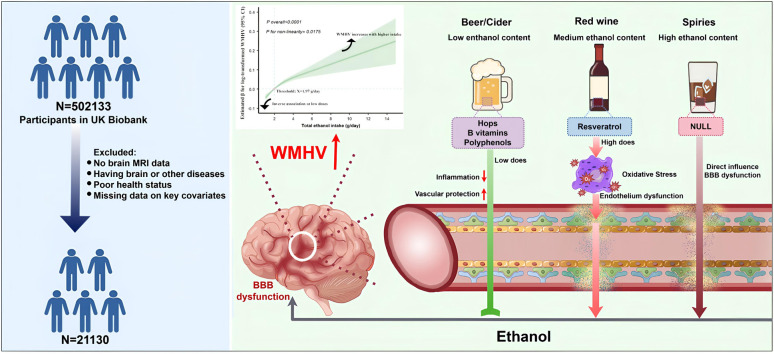

## 1 Introduction

Cerebral small vessel disease (CSVD) refers to a spectrum of pathological processes affecting the small arteries, arterioles, capillaries, and venules of the brain. It accounts for up to 25 % of all ischemic strokes and is the leading cause of lacunar stroke and spontaneous intracerebral hemorrhage. However, the clinical significance of CSVD extends far beyond stroke. It is increasingly recognized as the most common vascular pathology underlying vascular cognitive impairment (VCI), and a major contributor to Alzheimer’s disease, particularly in older adults [1]. The typical MRI hallmarks of CSVD include white matter hyperintensities (WMH), lacunes, cerebral microbleeds, enlarged perivascular spaces, and cortical atrophy [1]. These markers not only reflect cumulative microvascular injury but also predict functional outcomes such as cognitive decline, gait disturbance, depression, and apathy [2]. To better quantify the overall burden of CSVD, a composite measure known as the total CSVD score has been proposed, integrating the presence of these imaging markers into a single ordinal scale. This score has shown strong associations with cognitive impairment, functional disability, and dementia risk in both clinical and population-based studies [3,4]. Epidemiological studies report a high prevalence of radiological CSVD in both community-dwelling elderly individuals and patients with dementia, with WMH present in over 60 % of older adults and nearly 90 % of those with dementia. Notably, longitudinal studies have found that greater WMHV burden is associated with accelerated progression from mild cognitive impairment (MCI) to Alzheimer’s disease, underscoring CSVD as a key modifiable substrate in dementia prevention [4]. As such, identifying and modifying lifestyle-related risk factors that contribute to CSVD has emerged as a critical strategy to reduce the burden of late-life cognitive disorders.

Alcohol consumption has long been investigated as a potential determinant of cardiovascular and cerebrovascular health. However, the relationship between alcohol intake and cerebrovascular outcomes, including CSVD, remains controversial and complex. Some studies propose a dose-dependent association, where moderate alcohol consumption is linked to a reduced risk of cardiovascular and cerebrovascular diseases, likely due to its effects on lipid metabolism and anti-inflammatory pathways [5,6]. Conversely, excessive alcohol consumption is consistently associated with elevated blood pressure, atherosclerosis, and increased risk of stroke and cerebrovascular events [7]. A large prospective study of 512,715 Chinese patients concluded that alcohol consumption uniformly raises blood pressure and stroke risk, challenging the notion of a protective effect from moderate drinking [8]. These inconsistencies in findings may stem from methodological differences, confounding factors such as reverse causality, genetic predisposition, and varying definitions of alcohol consumption [9].

Importantly, alcohol consumption is a well-established risk factor for hypertension, atherosclerosis, and cardiomyopathy, conditions that also contribute to the development and progression of CSVD [10,11]. Notably, evidence suggests a positive association between alcohol consumption and the burden of white matter hyperintensities, one of the most significant MRI indicators of CSVD [12]. However, most of these studies have focused on total alcohol consumption without considering the potential heterogeneity in the effects of specific alcoholic beverages and drinking patterns.

Different alcoholic beverages, such as beer, wine, and spirits, contain distinct bioactive components that may influence cerebrovascular health in diverse ways. For example, polyphenolic antioxidants, such as resveratrol in wine, have been shown to exert protective effects against cardiovascular diseases by reducing oxidative stress, enhancing endothelial function, and exerting anti-inflammatory effects [13]. Similarly, hops-derived compounds in beer have been linked to antioxidant properties, which help mitigate vascular inflammation and oxidative damage [14]. In contrast, spirits—often lacking these protective polyphenolic compounds—are primarily composed of ethanol, which has been associated with higher risks of hypertension, atherosclerosis, and endothelial dysfunction [15]. This heterogeneity in bioactive content raises the question of whether the type and frequency of alcohol consumption may differentially impact CSVD burden. Specifically, the potential for resveratrol and hops-derived polyphenols to reduce oxidative stress and inflammation could result in protective effects on the blood-brain barrier and cerebral vasculature, while ethanol in spirits may exacerbate microvascular damage. Understanding how these beverage-specific components influence CSVD pathophysiology is critical to better understanding alcohol as a modifiable risk factor for CSVD and informing targeted prevention strategies.

Identifying drinking behaviors that are associated with higher or lower CSVD burden can provide valuable insights into the pathophysiology of CSVD and inform evidence-based recommendations for individual health management and public health policies. This study uses a large-scale prospective cohort data from the UK Biobank (UKB) to explore the relationship between total and specific alcoholic beverage consumption (including beer, wine, and spirits) and CSVD burden.

## 2 Methods

### 2.1 Data source and population

The study population consisted of participants who were part of the MRI component of the UKB project. UKB is an ongoing prospective cohort study that recruited over 500,000 volunteers aged 40 to 69 years from across the UK between 2006 and 2010, registering them at one of 22 research centers in the country. At baseline recruitment, each participant completed a standardized questionnaire, underwent a standardized interview with a study nurse, and had physical and physiological measurements taken, with blood, saliva, and urine samples collected [16]. The study was expanded in 2014 to initiate the world's largest multimodal imaging research, inviting 100,000 participants to undergo brain, cardiac, and abdominal MRI scans. Between 2014 and 2020, 42,806 participants underwent brain MRI scans [17]. The study was reported according to the *Strengthening the Reporting of Observational studies in Epidemiology* (STROBE) guidelines

### 2.2 Exclusion criteria

eFig. 1 illustrates the sample inclusion process for this study, with varying sample sizes across different analyses. Among the 502,133 participants originally enrolled in the cohort, 44,994 underwent brain MRI scans. Participants reporting diagnoses of dementia, multiple sclerosis, Parkinson’s disease, or any other chronic degenerative neurological disorder, including brain cancer, brain hemorrhage, brain abscess, aneurysm, cerebral palsy, encephalitis, head or neurological injury, nervous system infection, trauma, stroke, coronary heart disease, or chronic renal failure were excluded (*n* = 5,110), as assessed through a verbal interview with a trained nurse at the Assessment Center [18]. An additional 612 participants who self-reported poor health status at baseline were also excluded [19]. Furthermore, during the data cleaning process, participants were excluded if any of the variables used in the analysis contained responses such as “I do not know,” “Prefer not to answer,” or were missing; this resulted in the exclusion of a further 11,946 individuals. Consequently, a total of 27,326 participants were included in the preliminary analysis, among whom 21,130 were current drinkers with complete data on alcohol intake.

### 2.3 Standard protocol approvals, registrations, and patient consents

The UKB was approved by the Northwest Multi-Center Research Ethics Committee as a research tissue bank (21/NW/0157). All participants provided written informed consent to participate in the UK Biobank.

### 2.4 Assessment of alcohol consumption

Baseline alcohol consumption data were obtained using a computer-assisted touchscreen system. Participants were asked to report their drinking status as "never," "former," or "current." For individuals who consume alcohol, weekly drinking frequency was further assessed (1–2, 3–4, and ≥5 times per week), along with the average weekly consumption of each type of alcoholic beverage (red wine, champagne or white wine, beer or cider [including bitter, lager, strong beer, and Guinness, all containing alcohol], spirits, and fortified wine) [14]. Specific alcohol beverage intake was categorized into five groups: 0, ≤1, 2–3, 4–6, and ≥7 drinks per week for red wine, one glass of champagne or white wine, one pint of beer or cider, spirits, or one glass of fortified wine. To account for variations in ethanol content and beverage volume, the UKB’s definition of alcohol units was used: one pint or can of beer/lager/cider = 2 units; one 25 mL shot of spirits = 1 unit; one glass of wine = 1.5 units (eTable 1) [18,20]. The ethanol content in specific alcoholic beverages (in grams per day) was calculated, and the total ethanol consumption per day for alcohol drinkers was determined by summing the ethanol content from each type of alcoholic beverage.

### 2.5 Brain MRI acquisition and processing

All brain MRI data in the UKB were acquired using the same 3T Siemens Skyra scanner (Siemens Healthineers, Erlangen, Germany) according to the freely available protocol [21]. In this study, we used total white matter hyperintensity volume (WMHV) as a biomarker for the overall burden of cerebral small vessel disease. We utilized preprocessed T2-FLAIR-derived WMHV and intracranial volume generated by the image processing pipeline developed and operated by the UK Biobank [18]. Detailed information on T2-FLAIR image processing is provided in the study by Alfaro-Almagro et al. Briefly, FreeSurfer was used for brain tissue segmentation and quantification, and quality control procedures were applied to remove scans with motion artifacts or poor signal quality. The Brain Intensity Abnormality Classification Algorithm is a fully automated supervised method used to estimate total WMHV [22]. The WMHV data used in this study were directly sourced from the preprocessed UKB dataset, which was normalized by intracranial volume (WMHV_Norm_) to correct for variations in brain size across individuals.

### 2.6 Covariates

Covariates were selected based on previous studies, and these were obtained from comprehensive physical and clinical assessments of participants at baseline, supplemented by data from a computer touchscreen questionnaire. Demographic and socioeconomic variables included sex, ethnicity, education level, age at the time of the questionnaire, and the Townsend Deprivation Index (TDI) at recruitment. Ethnicity was classified into White or Others (include Mixed, Asian or Asian British, Black or Black British, Chinese, and Others). Education level was categorized as university degree or higher versus below university, and served as an individual-level indicator of socioeconomic status. The TDI score, representing an area-level indicator of material deprivation, is a composite measure based on four key variables: unemployment rate, overcrowded housing, non-car ownership, and non-home ownership. These two complementary measures were included to comprehensively account for socioeconomic status (SES) in our analysis [23].

Baseline blood biochemical measurements, including estimated glomerular filtration rate (eGFR), high-density lipoprotein cholesterol (HDL-C), and glycated hemoglobin (HbA1c), were obtained. eGFR was calculated using the Chronic Kidney Disease Epidemiology Collaboration (CKD-EPI) equation [24]. In addition, physical activity levels, family history of cardiovascular disease, and self-reported dietary intake of fruits and vegetables were assessed and included as covariates. Beneficial physical activity was defined according to the World Health Organization (WHO) guidelines as engaging in at least 150 min per week of moderate-intensity physical activity, or at least 75 min per week of vigorous-intensity physical activity, or an equivalent combination of both intensities exceeding 150 min weekly [25]. Family history of cardiovascular disease was determined based on self-reported parental history of myocardial infarction, stroke, or coronary artery disease. Additionally, self-reported intake of fruits and vegetables was included].

Cardiovascular risk factors included diabetes, hyperlipidemia, hypertension. Baseline diagnoses of diabetes, hyperlipidemia, and hypertension were determined based on participants' self-reported medical history, use of treatment medications during touchscreen and verbal interviews.

### 2.7 Statistical analysis

Baseline participant characteristics were summarized according to drinking frequency for current drinkers. Continuous variables were expressed as means and standard deviations (SD), while categorical variables were expressed as number and percentages. All variables were tested for normality using the Shapiro-Wilk test and confirmed to be normally distributed except for WMHV_Norm_, which required log-transformation. Transformed WMHV approximated normal distribution (eFigure2). Spearman's partial correlation coefficients were used to assess the correlation between specific alcoholic beverages.

To minimize potential confounding factors, we constructed multiple linear regression models with increasing covariate adjustments to precisely investigate the association between alcohol consumption and CSVD burden (evaluating Beta values and their 95 % confidence intervals). Model 1 was adjusted for age, sex, ethnicity, TDI and education level; Model 2 was further adjusted for physical activity, CVD family history, intake of fruits, intake of vegetables, eGFR, HDL-C and HbA1c; Model 3 was further adjusted for cardiovascular risk factors on the basis of Model 2, including diabetes, hypertension, and hyperlipidemia. In the preliminary analysis, we investigated the association between current drinking status and CSVD burden, with former and never drinkers classified as non-current drinkers. We then performed a sensitivity analysis excluding former drinkers. Subsequently, we selected current drinkers for the main analysis. First, we examined the association between their drinking frequency and CSVD burden and reported variance inflation factors (VIF) for covariates in Model 3 (eTable3). Next, we further analyzed the association between the consumption frequency of different types of alcoholic beverages and CSVD burden, with Benjamini-Hochberg adjustment for significance (eTable4). In addition, we assessed the relationship between total or beverage-specific ethanol consumption and CSVD burden among alcohol consumers, and visualized the results using restricted cubic splines (RCS). Subgroup analyses were also performed to evaluate the robustness of the association between drinking frequency and CSVD burden.

All statistical analyses were performed using R (version 4.3.1). A two-sided p-value of less than 0.05 was considered statistically significant.

## 3 Results

### 3.1 Baseline characteristics

The baseline characteristics of participants included in the main analysis are summarized in Table 1. There were 211,30 participants included for the main analysis. Among them (mean [SD] age, 54.59 [7.35] years; 98 % White participants, 1.6 % participants of other ethnicity), 10,447 (49 %) were females and 10,683 (51 %) were males. Among them, 6967 (33 %) were reported drinking 1–2 times/week; 7910 (37 %) were reported drinking alcohol 3–4 times/week and 6253 (30 %) drinking alcohol ≥5 times/week. Participants drinking ≥5 times/week had higher CSVD burden than those who drinking 1–2 times/week or 3–4 times/week. Participants drinking ≥5 times/week were also more likely to have hyperlipidemia or hypertension, and exhibited lower TDI scores, indicative of higher socioeconomic status.

**Table 1:** Baseline Characteristics of Participants.

Characteristic	Overall, *N* = 21,130(100 %)^1^	1–2/week *N* = 6967(33 %)	3–4/week *N* = 7910(37 %)	>5/week *N* = 6253(30 %)	P-Value
Age (years)	54.59 ± 7.35	53.40 ± 7.34	54.40 ± 7.22	56.15 ± 7.24	<0.001
Gender (%)					<0.001
Female	10,447(49 %)	3825(55 %)	3910(49 %)	2712(43 %)	
Male	10,683(51 %)	3142(45 %)	4000(51 %)	3541(57 %)	
Ethnicity (%)					<0.001
White	20,789(98 %)	6820(98 %)	7797(99 %)	6172(99 %)	
Others	341(1.6 %)	147(2.1 %)	113(1.4 %)	81(1.3 %)	
Education status					<0.001
Less than college	10,009(47 %)	3727(53 %)	3670(46 %)	2612(42 %)	
College	11,121(53 %)	3240(47 %)	4240(54 %)	3641(58 %)	
TDI	−2.09 ± 2.59	−2.05 ± 2.60	−2.20 ± 2.56	−1.99 ± 2.63	<0.001
Healthy physical activity					0.002
No	8844(42 %)	2929(42 %)	3203(40 %)	2712(43 %)	
Yes	12,286(58 %)	4038(58 %)	4707(60 %)	3541(57 %)	
CVD family history					
No	8179(39 %)	2780(40 %)	3092(39 %)	2307(37 %)	
Yes	12,951(61 %)	4187(60 %)	4818(61 %)	3946(63 %)	
eGFR (ml/min/1.73 m^2^)	94 ± 13	94 ± 13	95 ± 13	93 ± 13	<0.001
HDL-C (mmol/L)	1.52 ± 0.38	1.45 ± 0.36	1.52 ± 0.37	1.58 ± 0.40	<0.001
HbA1c (%)	5.32 ± 0.42	5.32 ± 0.43	5.31 ± 0.39	5.32 ± 0.45	0.4
Vegetables(tablespoons/day)	5 ± 3	5 ± 3	5 ± 3	5 ± 3	0.003
Fruit (pieces/day)	3 ± 2	3 ± 2	3 ± 2	3 ± 2	<0.001
Diabetes					0.2
No	20,883(88 %)	6877(99 %)	7831(99 %)	6175(99 %)	
Yes	247(1.2 %)	90(1.3 %)	79(1.0 %)	78(1.2 %)	
Hypertension					<0.001
No	18,587(88 %)	6202(89 %)	6999(88 %)	5386(86 %)	
Yes	2543(12 %)	765(11 %)	911(12 %)	867(14 %)	
Hyperlipidemia					<0.001
No	5079(24 %)	1878(27 %)	1926(24 %)	1275(20 %)	
Yes	16,051(76 %)	5089(73 %)	5984(76 %)	4978(80 %)	
WMHV_Norm_ (logit)	−6.22 ± 1.02	−6.32 ± 1.01	−6.24 ± 1.02	−6.09 ± 1/02	<0.001

1 mean (IQR) for continuous; n (%) for categorical.

2 Kruskal-Wallis rank sum test; Pearson’s Chi-squared test.

### 3.2 Alcohol consumption and CSVD burden

In the preliminary analysis, we first examined the association between current drinking status and CSVD burden using non-current drinkers as the reference group (Table 2). In the fully adjusted Model 3, Current alcohol consumption was independently associated with greater CSVD burden (β = 0.07, 95 % CI: 0.00–0.15). Since we used log-transformed WMHV, this corresponds to approximately a 7.25 % higher mean WMHV in drinkers compared to non-drinkers. The results of sensitivity analysis excluding former drinkers were consistent with the results (eTable2).

**Table 2:** Association Between Current Drinking Status and CSVD Burden.

Group	Characteristic	Beta	95 % CI^1^	p-value
Model1	Non-current drinkers	Ref		
	Current drinkers	0.06	−0.02, 0.13	0.122
Model2	Non-current drinkers	Ref		
	Current drinkers	0.08	0.01, 0.15	0.030
Model3	Non-current drinkers	Ref		
	Current drinkers	0.07	0.00, 0.15	0.046

Abbreviations: CSVD, cerebral small vessel disease; Ref, reference (Beta=0); wk, week.

Model 1 was adjusted for age, sex, ethnicity, TDI, education level.

Model 2 was additionally adjusted for healthy physical activity, CVD family history, intake of fruits, intake of vegetables, eGFR, HDLC and HbA1c.

Model 3 was additionally adjusted for diabetes, hypertension and hyperlipidemia.

AIC values for the models: Model 1: 70,661.12; Model 2: 70,541.53; Model 3: 70,357.77.

Cohen’s d in Model 3: 0.1.

### 3.3 Frequency of alcohol consumption and CSVD burden

**Fig. 1:**
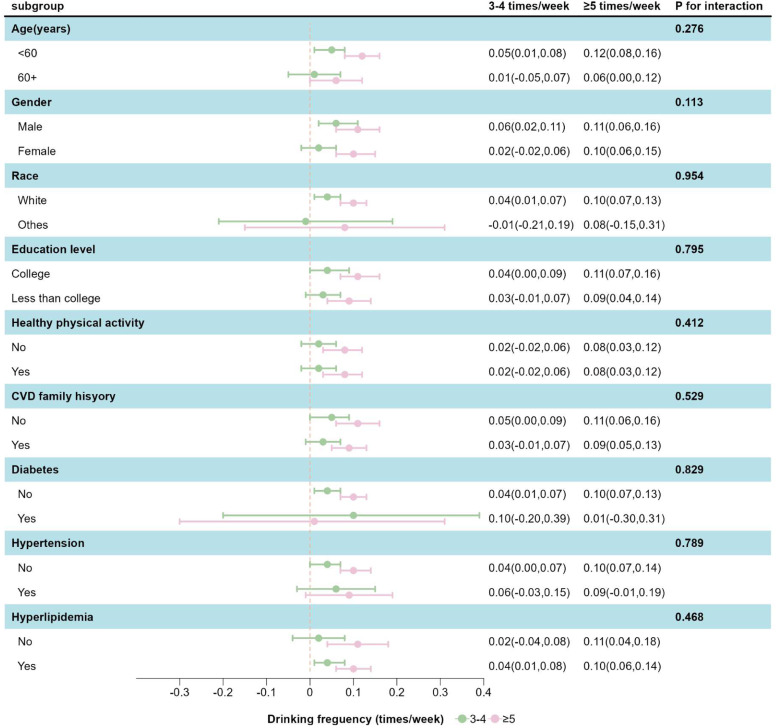
Subgroup Analysis of the Association between Frequency of Alcohol Consumption and CSVD Burden. Abbreviations: CSVD, cerebral small vessel disease. Result for analysis was adjusted for age, sex, ethnicity, TDI, education level, healthy physical activity, CVD family history, intake of fruits, intake of vegetables, eGFR, HDL-C, HbA1c, diabetes, hypertension and hyperlipidemia.

Table 3 presents the relationship between alcohol consumption frequency and CSVD burden. In the main analysis, higher CSVD burden was associated with more frequent alcohol intake. Specifically, compared to drinking 1–2 times/week, drinking 3–4 times/week was associated with a modest increase in CSVD burden (β = 0.04, 95 % CI: 0.01–0.07), and drinking ≥ 5 times/week showed a stronger association (β = 0.10, 95 % CI: 0.07–0.13). Subgroup analyses (Fig. 1) showed no significant interactions, indicating consistent findings across subgroups. eTable3 showed no multicollinearity among model 3 covariates.

**Table 3:** Association between Alcohol Consumption Frequency and CSVD Burden Among Current Drinkers.

Model	Risk of CSVD, Beta (95 % CI)			P for trend
	1–2 drinks/wk	3–4 drinks/wk	≥5 drinks/wk	
Model 1	Ref.	0.03 (0.00,0.06)	0.10 (0.06,0.13)	<0.001
Model 2	Ref.	0.04 (0.01,0.07)	0.11 (0.08,0.14)	<0.001
Model 3	Ref.	0.04 (0.01,0.07)	0.10 (0.07,0.13)	<0.001

Abbreviations: CSVD, cerebral small vessel disease; Ref, reference (Beta=0); wk, week.

Model 1 was adjusted for age, sex, ethnicity, TDI, education level.

Model 2 was additionally adjusted for healthy physical activity, CVD family history, intake of fruits, intake of vegetables, eGFR, HDLC and HbA1c.

Model 3 was additionally adjusted for diabetes, hypertension and hyperlipidemia.

AIC values for the models: Model 1: 57,246.19; Model 2: 56,513.61; Model 3: 56,219.64.

### 3.4 Consumption of specific alcoholic beverages and the burden of CSVD

The correlation between specific alcoholic beverages was generally weak (eFig. 2). eFigure 3 shows the weekly average consumption of specific alcoholic beverages among current drinkers in the main analysis. Red wine was the most commonly consumed, with an average of 4.46 glasses per week, while fortified wine was the least consumed, with an average of 0.22 glasses per week. We analyzed the association between the consumption of different alcoholic beverages and CSVD burden in alcohol drinkers (Table 4). Notably, moderate-to-low consumption of beer and cider (≤3 servings/week) was significantly negatively associated with CSVD burden. These findings highlight the differential effects of alcoholic beverages on CSVD burden, emphasizing the importance of beverage type when assessing alcohol-related cerebrovascular outcomes. eTable4 showed that the significance of the observed associations remained robust after adjustment for Benjamini-Hochberg.

**Table 4:** Association between Specific Alcoholic Beverages Consumption and CSVD Burden Among Current Drinkers.

Alcoholic beverage	CSVD Burden, Beta (95 % CI)					
	0 drinks/wk	≤1drinks/wk	2–3drinks/wk	4–6drinks/wk	≥7 drinks/wk	P for trend
Red wine, glasses/wk						
Model1	Ref	−0.01 (−0.06, 0.04)	−0.01 (−0.04, 0.03)	0.04 (0.00, 0.07)	0.05 (0.01, 0.08)	0.004
Model2	Ref	−0.02 (−0.07, 0.02)	−0.01 (−0.04, 0.03)	0.04 (0.00, 0.08)	0.06 (0.02, 0.09)	<0.001
Model3	Ref	−0.02 (−0.07, 0.03)	−0.01 (−0.04, 0.03)	0.03 (0.00, 0.07)	0.04 (0.01, 0.08)	0.004
Champagne/White wine, glasses/wk						
Model1	Ref	−0.03 (−0.07, 0.01)	0.01 (−0.02, 0.05)	0.02 (−0.02, 0.05)	0.03 (−0.01, 0.08)	0.082
Model2	Ref	−0.03 (−0.07,0.01)	0.01 (−0.02,0.05)	0.02 (−0.02,0.06)	0.05 (0.00,0.09)	0.019
Model3	Ref	−0.03 (−0.07,0.01)	0.01 (−0.02,0.05)	0.02 (−0.01,0.06)	0.04 (0.00,0.09)	0.021
Beer/Cider, pints/wk						
Model1	Ref	−0.09 (−0.13, −0.05)	−0.10 (−0.14, −0.06)	−0.05 (−0.10, −0.01)	0.02 (−0.03, 0.07)	0.407
Model2	Ref	−0.09 (−0.13, −0.05)	−0.08 (−0.12, −0.04)	−0.04 (−0.08, 0.01)	0.04 (−0.01,0.09)	0.773
Model3	Ref	−0.08 (−0.12, −0.04)	−0.08 (−0.12, −0.04)	−0.03 (−0.08, 0.02)	0.04 (−0.01, 0.09)	0.622
Spirits, measures/wk						
Model1	Ref	−0.02 (−0.06, 0.02)	0.09 (0.05, 0.13)	0.11 (0.06, 0.16)	0.17 (0.11, 0.23)	<0.001
Model2	Ref	−0.03 (−0.07, 0.01)	0.08 (0.04, 0.12)	0.10 (0.05, 0.15)	0.15 (0.09, 0.21)	<0.001
Model3	Ref	−0.03 (−0.07, 0.01)	0.08 (0.04, 0.11)	0.10 (0.05, 0.14)	0.13 (0.07, 0.19)	<0.001
Fortified wine, glasses/wk						
Model1	Ref	−0.01 (−0.07, 0.04)	0.06 (−0.01, 0.13)	0.02 (−0.01, 0.15)	0.13 (−0.08, 0.34)	0.147
Model2	Ref	−0.03 (−0.08, 0.02)	0.04 (−0.03, 0.11)	0.01 (−0.11, 0.14)	0.12 (−0.09, 0.32)	0.441
Model3	Ref	−0.03 (−0,08, 0.02)	0.04 (−0.03, 0.11)	0.02 (−0.11, 0.14)	0.11 (−0.10, 0.31)	0.429

Abbreviations: CSVD, cerebral small vessel disease; Ref, reference (Beta=0); wk,week.

Model 1 was adjusted for age, sex, ethnicity, TDI, education level.

Model 2 was additionally adjusted for healthy physical activity, CVD family history, intake of fruits, intake of vegetables,.

eGFR, HDLC and HbA1c.

Model 3 was additionally adjusted for diabetes, hypertension and hyperlipidemia.

AIC values for the models in Red wine group: Model 1: 57,272.57; Model 2: 56,541.06; Model 3: 56,246.86.

AIC values for the models in Champagne/White wine group: Model 1: 57,278.97; Model 2: 56,549.01; Model 3: 56,250.19.

AIC values for the models in Beer/Cider group: Model 1: 57,240.81; Model 2: 56,513.07; Model 3: 56,219.27.

AIC values for the models in Spirits group: Model 1: 57,221.13; Model 2: 56,502.59; Model 3: 56,211.42.

AIC values for the models in Spirits group: Model 1: 57,280.56; Model 2: 56,555.84; Model 3: 56,256.53.

### 3.5 Pure ethanol intake and CSVD burden

We further assessed the relationship between ethanol content in specific alcoholic beverages and CSVD burden, with results consistent with the beverage-specific analysis (eFigure 4). For each 1 g/day increase in total ethanol intake, CSVD burden increased by 3 % (Beta = 0.03; 95 % CI, 0.02–0.03).

**Fig. 2:**
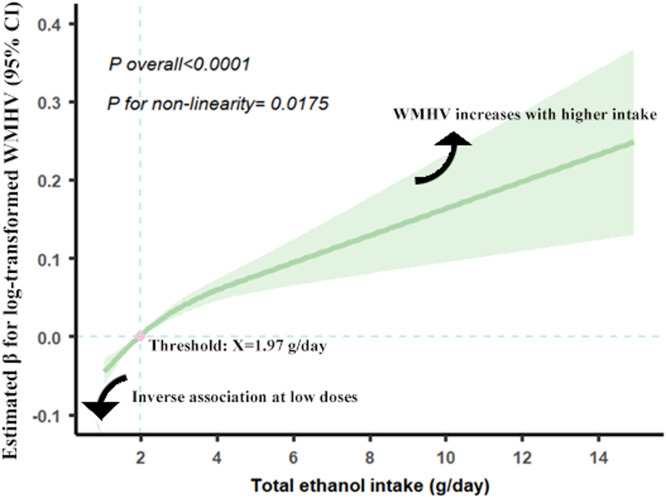
Restricted Cubic Splines for the Relationship between Total Ethanol Intake and CSVD Burden. Abbreviations: CSVD, cerebral small vessel disease. Result for analysis was adjusted for age, sex, ethnicity, TDI, education level, healthy physical activity, CVD family history, intake of fruits, intake of vegetables, eGFR, HDL-C, HbA1c, diabetes, hypertension and hyperlipidemia.

In addition, RCS analysis showed a significant non-linear association between ethanol intake and CSVD burden (P for non-linearity = 0.0175) (Fig. 2). At lower ethanol consumption levels (<1.97 g/day), there was a mild negative association, as indicated by negative Beta values. However, beyond this threshold, CSVD burden increased in a dose-dependent manner.

## 4 Discussion

In this large-scale study based on cross-sectional data from UKB, we systematically evaluated the associations of alcohol consumption—including drinking status, drinking frequency, overall ethanol consumption, and specific beverage types—with CSVD burden, as measured by normalized WMHV. We observed that alcohol consumers exhibited significantly higher CSVD burden compared to non-current drinkers after comprehensive adjustment for confounders. Among alcohol drinkers, increased drinking frequency was associated with progressively greater CSVD burden, highlighting a dose-response relationship. Additionally, our analyses demonstrated distinct effects of specific alcoholic beverages; moderate-to-high intake of red wine, white wine/champagne, and spirits correlated positively with increased CSVD burden, while moderate-to-low consumption of beer and cider appeared to have an inverse association. Furthermore, restricted cubic spline analyses indicated a significant non-linear relationship between total ethanol consumption and CSVD burden, revealing a mild negative association at very low intake levels (<1.97 g/day), followed by a marked detrimental effect with increasing ethanol consumption. Collectively, these findings underscore the complexity of alcohol's effects on cerebrovascular health, emphasizing the importance of both drinking patterns and beverage types in determining CSVD risk.

In our preliminary analysis, after adjustment for comprehensive covariates, alcohol consumers had an average WMHV approximately 7.25 % higher than non-drinkers. However, when evaluating the magnitude of these associations using effect size measures, we found that the effect of drinking status on CSVD burden, as indicated by Cohen's d, was relatively small (Cohen’s *d* = 0.1 for Model 3). This suggests a limited association between alcohol consumption and CSVD burden. While this difference may not be clinically meaningful at the individual level, it could carry public health relevance given the high prevalence of alcohol consumption and the cumulative nature of CSVD. Therefore, although we interpret these findings with caution, the observed association between alcohol intake and increased CSVD burden remains significant in the context of population-level cerebrovascular health. This finding aligns with previous studies suggesting an overall adverse effect of current drinking on cerebral small vessel health. For instance, a recent population-based study also observed increased WMHV among alcohol consumers compared to abstainers, supporting our findings [26]. However, it should be noted that some epidemiological evidence presents inconsistent findings, where moderate alcohol intake has been suggested to exert potential protective effects against cerebrovascular changes, possibly due to alcohol-induced improvements in lipid metabolism and anti-inflammatory pathways [27]. These discrepancies may result from variations in methodological approaches, population characteristics, and definitions of alcohol consumption across studies.

The present study revealed that higher alcohol consumption frequency is strongly associated with increased CSVD burden. Compared to individuals who drank 1–2 times per week, those drinking 3–4 times per week exhibited a modest increase in risk, while participants consuming alcohol ≥5 times per week demonstrated a more pronounced association with CSVD. This dose-dependent association aligns with previous studies linking frequent alcohol consumption to WMH and lacunar infarcts [1,12]. The positive association between frequent alcohol intake and CSVD burden may be attributable to the cumulative toxic effects of ethanol on small vessel integrity. Chronic ethanol exposure can contribute to endothelial dysfunction, hypertension, and oxidative stress—key mechanisms implicated in the pathogenesis of CSVD [1,10].

Even after adjusting for self-reported hypertension and hyperlipidemia in Model 3, higher drinking frequency remained significantly associated with greater CSVD burden. Since both conditions are established cerebrovascular risk factors and showed increasing prevalence with higher alcohol consumption in our baseline data, this persistent association suggests that alcohol may affect CSVD through mechanisms beyond its vascular effects. Potential pathways include direct endothelial toxicity from ethanol or its metabolites, disruption of the blood–brain barrier, and promotion of oxidative stress and inflammation [28,29]. Nevertheless, the use of self-reported data for vascular comorbidities may have introduced misclassification or underreporting, potentially leading to residual confounding. Future studies incorporating objective clinical measurements and longitudinal follow-up are needed to validate these findings and elucidate the underlying mechanisms.

Our analysis of specific alcoholic beverages revealed differential associations with CSVD burden. Moderate-to-high consumption of red wine, spirits, white wine, and champagne was positively associated with greater CSVD burden, whereas moderate-to-low consumption of beer and cider demonstrated a significant negative association. These findings emphasize the need to consider the type of alcoholic beverage when assessing alcohol's cerebrovascular effects.

The observed association between red wine and CSVD burden is somewhat surprising, given prior studies suggesting potential cardiovascular benefits of moderate red wine consumption [30], especially for coronary heart disease, atrial fibrillation, and myocardial infarction [[31], [32], [33]], due to its polyphenolic antioxidant content [13,14]. However, such protective effects may largely be attributed to the fact that red wine consumption is often associated with higher levels of education, income, and healthier lifestyle behaviors, such as better diet, regular physical activity, and non-smoking [34]. Thus, our finding that these benefits may not extend to small vessel health is not entirely surprising particularly at moderate-to-high intake levels. The detrimental effects of ethanol itself may overshadow any protective properties of polyphenols when alcohol consumption exceeds a certain threshold. In contrast, the negative association between moderate-to-low beer and cider consumption and CSVD burden warrants further investigation. Beer and cider may contain bioactive compounds, such as polyphenols and B vitamins, that could exert protective effects on cerebrovascular health at low consumption levels [[35], [36], [37]]. This observation is consistent with prior evidence suggesting that certain components in beer may promote endothelial function and reduce inflammation [13]. It is essential to emphasize that this association was observed only at low intake levels (≤1–3 servings/week), and excessive beer or cider consumption is likely to negate any potential benefits. However, non-physiological factors may also contribute to this inverse association. Studies have shown that moderate beer consumption is associated with better physical vitality, self-perception, psychological well-being, and social-emotional health, particularly among individuals from lower socioeconomic groups [38]. Therefore, the observed inverse association may partly reflect these underlying socioeconomic or lifestyle differences, rather than the inherent properties of the beverage itself. Accordingly, we interpret the potential protective effect of beer with caution. Additionally, no significant association was found between fortified wine consumption and CSVD burden. This null finding may reflect the low average consumption of fortified wine in the sample. Further studies in larger samples or among individuals with higher levels of fortified wine intake are needed to clarify its potential cerebrovascular impact.

The debate over whether alcohol consumption has a protective effect on cardiovascular and cerebrovascular health is ongoing [39]. To explore this discrepancy and eliminate potential biases caused by differences in the ethanol content of specific alcoholic beverages, we further analyzed the association between daily pure ethanol intake and CSVD burden in current drinkers. The results indicated a non-linear relationship. When daily ethanol intake was below 1.97 g, it was negatively associated with CSVD burden, but once this threshold was exceeded, any level of overall ethanol intake increased CSVD burden. Therefore, our study suggests that moderate alcohol consumption may not increase CSVD burden and may even have a mild alleviating effect, although further research is needed to confirm this hypothesis. Nonetheless, the observed threshold effect has important public health implications: it could help refine current drinking guidelines, especially for older populations at higher risk of cerebrovascular diseases such as stroke or vascular dementia. Studies have shown that over 60 % of older adults and nearly 90 % of dementia patients have WMHV burden, and an increase in WMHV burden accelerates the progression of MCI to Alzheimer's disease [4]. Therefore, controlling alcohol consumption to ≤2 g/day in these populations is critical for managing their condition and improving cerebrovascular health. The existence of this threshold further underscores the detrimental effects of heavy drinking (≥2 g/day) on brain health, supporting public health initiatives aimed at reducing the overall burden of CSVD and other alcohol-related health issues.

There are several limitations to this study. First, due to the cross-sectional nature of our data, the temporal sequence between alcohol exposure and the development of CSVD remains uncertain. This design cannot rule out reverse causation, whereby individuals with pre-existing brain pathology might alter their drinking behavior. Second, we excluded 11,946 participants due to missing covariates, introducing the possibility of selection bias. This could limit the representativeness of our analytic sample, potentially affecting the generalizability of our findings. Although multiple imputation methods could mitigate such bias, they were not feasible due to the extent and pattern of missing data. Third, assessments of alcohol consumption and baseline vascular comorbidities were based on self-report. This methodology may introduce recall bias and misclassification. For instance, heavy or frequent drinkers might underreport consumption due to social desirability, attenuating the observed associations. Conversely, occasional or former drinkers could be misclassified as non-drinkers, potentially diluting group differences. Similarly, self-reported diagnoses of diabetes, hyperlipidemia, and hypertension could lead to underreporting, overreporting, or omission of undiagnosed cases, causing residual confounding despite statistical adjustment. Fourth, psychiatric comorbidities such as depression and anxiety disorders—known to coexist frequently with alcohol use—were not available and thus not adjusted for in our analysis. Given the established interrelationship between psychiatric disorders and alcohol consumption and their direct association with increased WMHV burden [[40], [41], [42]], this omission may have influenced our results. Additionally, genetic factors such as APOE ε4 status, a well-established cerebrovascular risk factor associated with increased WMH burden and microvascular pathology, were not available for inclusion, further contributing to potential residual confounding [43]. Moreover, sleep quality may also affect the burden of CSVD. Studies have shown that poor sleep quality can increase the burden of CSVD [44]. Therefore, despite the extensive covariate adjustment in this study, due to the limitations of the data, it is inevitable that these confounding factors remain. Finally, while WMHV is a well-established imaging marker for assessing CSVD, it represents only one dimension of this multifaceted condition. Other important radiological markers such as lacunes, cerebral microbleeds, enlarged perivascular spaces, and brain atrophy were not included in our analyses due to data limitations. Thus, the single-marker approach we used may not fully capture the overall burden of CSVD. Future research should aim to overcome these limitations by adopting longitudinal cohort designs or Mendelian randomization to clarify causality. Furthermore, comprehensive adjustment for genetic, psychiatric factors, and sleep quality, objective measures of alcohol use and vascular health, and integration of multiple neuroimaging markers will strengthen causal inference and clinical relevance. Additionally, studies conducted in diverse and representative populations are warranted to improve generalizability and translational applicability.

## 5 Conclusion

In summary, our study highlights the complex relationship between alcohol consumption and CSVD burden. Higher drinking frequency, elevated total ethanol intake, and moderate-to-high consumption of specific alcoholic beverages—including red wine, spirits, and white wine—were significantly associated with increased CSVD burden. In contrast, lower consumption of beer and cider demonstrated a negative association, warranting further investigation to confirm these findings and elucidate the underlying mechanisms.

## Source of funding

This study was supported by National Natural Science Foundation of China (No. 82471368), Science and Technology Innovation Team of Anhui Province (No. 2024AH010014), Clinical and Translational Research Project of Anhui Province (No. 202427b10020090), and Research Fund of Anhui Institute of Translational Medicine (No. 2022zhyx-C38).

## Data availability

The UK Biobank data are available online at https://www.ukbiobank.ac.uk. All qualified researchers are able to apply for data used for the health-related research.

## Ethics approval and consent to participate

The UK Biobank study was approved by Northwest Multicentre Research Ethics Committee (21/NW/0157). All participants have provided written informed consent.

## Consent for publication

All authors read the manuscript and agreed to its publication.

## CRediT authorship contribution statement

**Ben-Bo Xiong:** Writing – original draft. **Zi-Jie Wang:** Formal analysis. **Zhi-Ming Li:** Writing – original draft. **Tian-Nan Yang:** Writing – review & editing. **Xiang-Yu Li:** Writing – review & editing. **Meng-Jie Lu:** Writing – review & editing. **Qi Li:** Writing – review & editing, Funding acquisition, Conceptualization.

## Declaration of competing interest

The authors declare that they have no known competing financial interests or personal relationships that could have appeared to influence the work reported in this paper.

## Appendix Supplementary materials

Image, application 1
