# Supplementary material for: Association between alcoholic beverage consumption and cerebral small vessel disease burden
Source: J Prev Alzheimers Dis. 2025 Aug 5;12(10):100322. doi: 10.1016/j.tjpad.2025.100322 (PMC12627891; doi:10.1016/j.tjpad.2025.100322)
Supplement: Supplementary file 1 [file mmc1.pdf]

Supplementary Material

Association Between Alcoholic Beverage Consumption and Cerebral Small Vessel Disease Burden

Table of contents

eTable 1. Alcohol Unit Definitions in UK Biobank

eTable 2. Association Between Current Drinking Status and CSVD Burden without Former Drinkers

eTable 3. VIFs for Covariates in the Analysis of Alcohol Consumption Frequency and CSVD Burden

eTable 4. Benjamini-Hochberg Adjusted P-values for Specific Alcoholic Beverages Consumption and CSVD Burden

eFigure 1. Flow Diagram of Participant Selection

eFigure 2. Distribution and normality check of log-transformed WMHV

eFigure 3. Spearman Partial Correlations Among Specific Alcoholic Beverages Among Current Drinkers

eFigure 4. Weekly Mean Consumption of Alcoholic Beverages Among Current Drinkers

eFigure 5. Pure Ethanol Intake (g/day) From Specific Alcoholic Beverages and CSVD Burden Among Current Drinkers

eTable 1. Alcohol Unit Definitions in UK Biobank

Alcoholic beverage type	Unit of measurement	Pure ethanol per measurement (unit)
Red wine	Glass (1/6 bottle)	1.5
White wine/Champagne	Glass (1/6 bottle)	1.5
Beer/Cider	Pint	2
Spirit	Measurement (1/25 bottle)	1
Fortified wine	Glass (1/12 bottle)	1

One unit of alcohol is defined as 10 mL (or 8 grams) of pure ethanol, according to UK Biobank definitions based on beverage type and typical alcohol by volume (ABV).

eTable 2. Association Between Current Drinking Status and CSVD Burden without Former Drinkers

Group	Characteristic	Beta	95% CI[1]	p-value
Model1	Non-current drinkers	Ref		
	Current drinkers	0.06	-0.01, 0.13	0.120
Model2	Non-current drinkers	Ref		
	Current drinkers	0.08	0.01, 0.15	0.030
Model3	Non-current drinkers	Ref		
	Current drinkers	0.07	0.00, 0.14	0.046

Abbreviations: CSVD, cerebral small vessel disease; Ref, reference (Beta=0); wk, week

Model 1 was adjusted for age, sex, ethnicity, TDI, education level.

Model 2 was additionally adjusted for healthy physical activity, CVD family history, intake of fruits, intake of vegetables, eGFR, HDLC and HbA1c.

Model 3 was additionally adjusted for diabetes, hypertension and hyperlipidemia.

eTable 3. VIFs for Covariates in the Analysis of Alcohol Consumption Frequency and CSVD Burden

Covariate	VIF Value
Age	1.257
Sex	1.363
Ethnicity	1.013
TDI	1.012
Education status	1.024
Healthy physical activity	1.042
CVD family history	1.032
Vegetables (tablespoons/day)	1.076
Fruit (pieces/day)	1.097
HDL-C (mmol/L)	1.447
HbA1c (%)	1.318
Diabetes	1.252
Hyperlipidemia	1.081
Hyperlipidemia	1.099

Abbreviations: CSVD, cerebral small vessel disease; VIF, variance inflation factors
Result for analysis was in Model3: adjusted for age, sex, ethnicity, TDI, education level, healthy physical activity, CVD family history, intake of fruits, intake of vegetables, eGFR, HDL-C, HbA1c, diabetes, hypertension and hyperlipidemia.

eTable 4. Benjamini-Hochberg Adjusted P-values for Specific Alcoholic Beverages Consumption and CSVD Burden

Alcoholic beverage	CSVD Burden, **Adjusted P-values**					
	0 drinks/wk	≤1 drinks/wk	2-3 drinks/wk	4-6 drinks/wk	≥7 drinks/wk	P for trend
Red wine, glasses/wk						
Model1	Ref	0.747	0.747	0.104	0.084	0.001
Model2	Ref	0.453	0.657	0.046	0.017	0.001
Model3	Ref	0.557	0.686	0.033	0.048	0.011
Champagne/White wine, glasses/wk						
Model1	Ref	0.405	0.479	0.479	0.405	0.137
Model2	Ref	0.173	0.406	0.330	0.155	0.032
Model3	Ref	0.262	0.384	0.303	0.189	0.036
Beer/Cider, pints/wk						
Model1	Ref	<0.001	<0.001	0.023	0.538	0.407
Model2	Ref	<0.001	<0.001	0.109	0.109	0.773
Model3	Ref	<0.001	<0.001	0.205	0.128	0.633
Spirits, measures/wk						
Model1	Ref	0.280	<0.001	<0.001	<0.001	<0.001
Model2	Ref	0.119	<0.001	<0.001	<0.001	<0.001
Model3	Ref	0.106	<0.001	<0.001	<0.001	<0.001
Fortified wine, glasses/wk						
Model1	Ref	0.708	0.424	0.708	0.424	0.186
Model2	Ref	0.365	0.365	0.828	0.365	0.551
Model3	Ref	0.407	0.407	0.793	0.407	0.536

Abbreviations: CSVD, cerebral small vessel disease; Ref, reference (Beta=0); wk,week

Model 1 was adjusted for age, sex, ethnicity, TDI, education level.

Model 2 was additionally adjusted for healthy physical activity, CVD family history, intake of fruits, intake of vegetables, eGFR, HDLC and HbA1c.

Model 3 was additionally adjusted for diabetes, hypertension and hyperlipidemia.

eFigure 1. Flow Diagram of Participant Selection

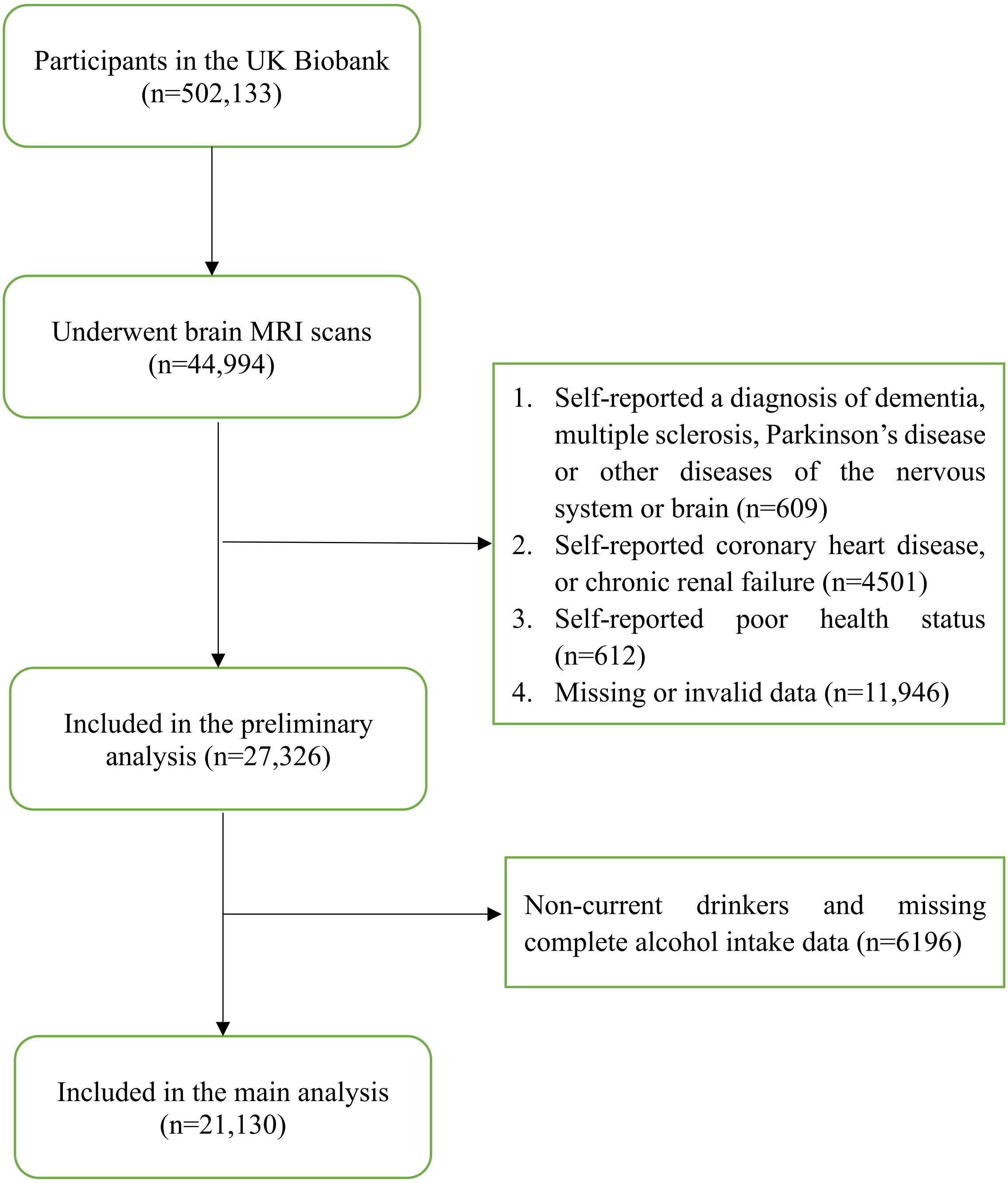

eFigure 2. Distribution and normality check of log-transformed WMHV.

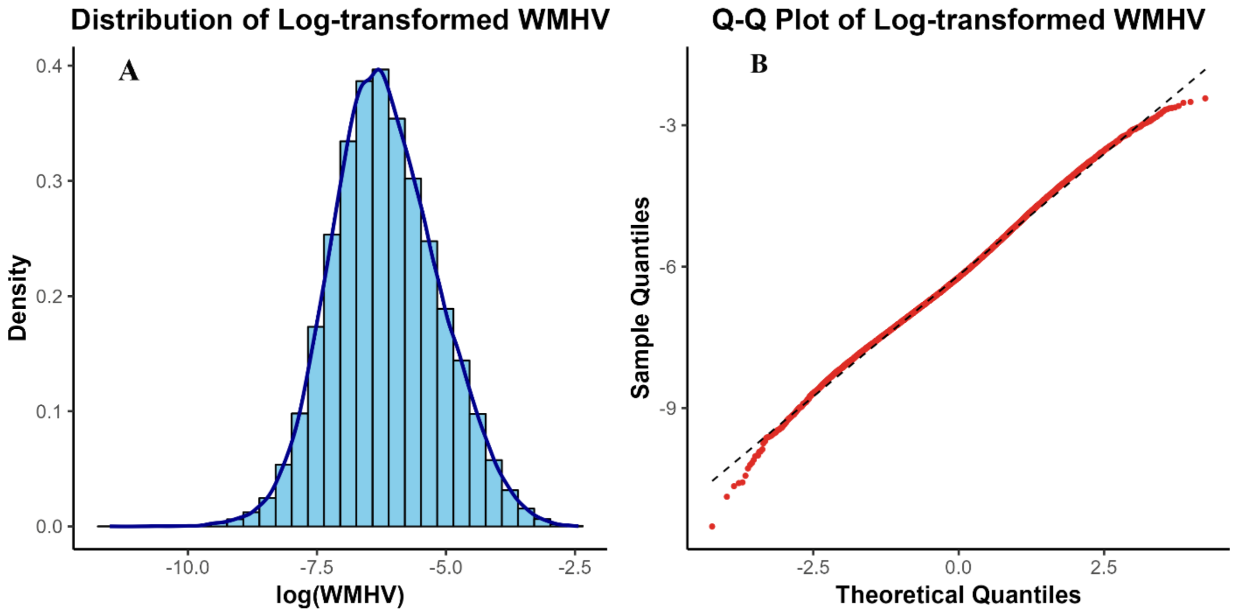

Abbreviations: WMHV, white matter hyperintensity volume

Panel A shows the histogram and density curve of log (WMHV), indicating an approximately normal distribution.

Panel B presents the corresponding Q-Q plot, supporting the use of linear regression models based on normality assumptions.

eFigure 3. Correlation matrix of weekly consumption across different types of alcoholic beverages.

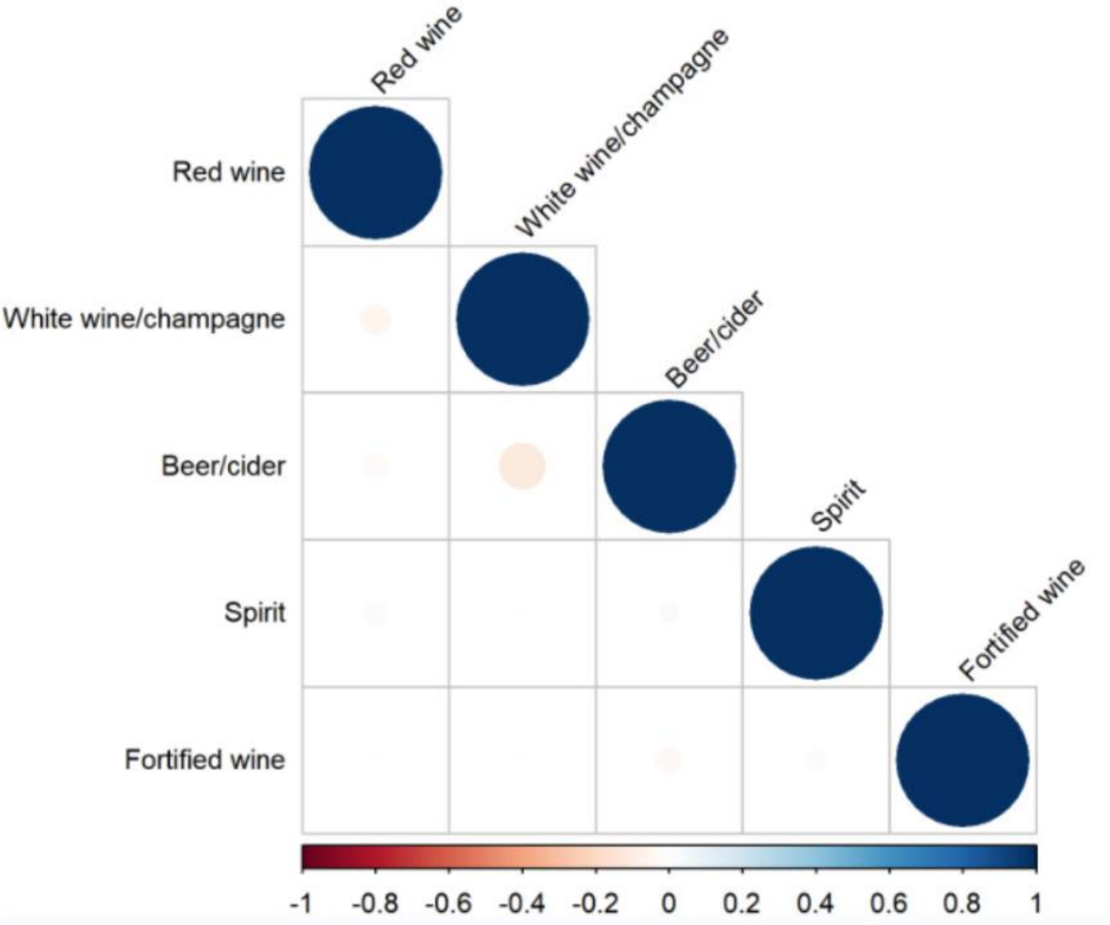

Note: The figure displays pairwise Pearson correlation coefficients between the average weekly consumption (in drinks/week) of six alcoholic beverage types among current drinkers. Color intensity and circle size reflect the strength and direction of the correlations. Dark blue indicates strong positive correlations, while red indicates negative correlations.

eFigure 4. Weekly Mean Consumption of Alcoholic Beverages Among Current Drinkers

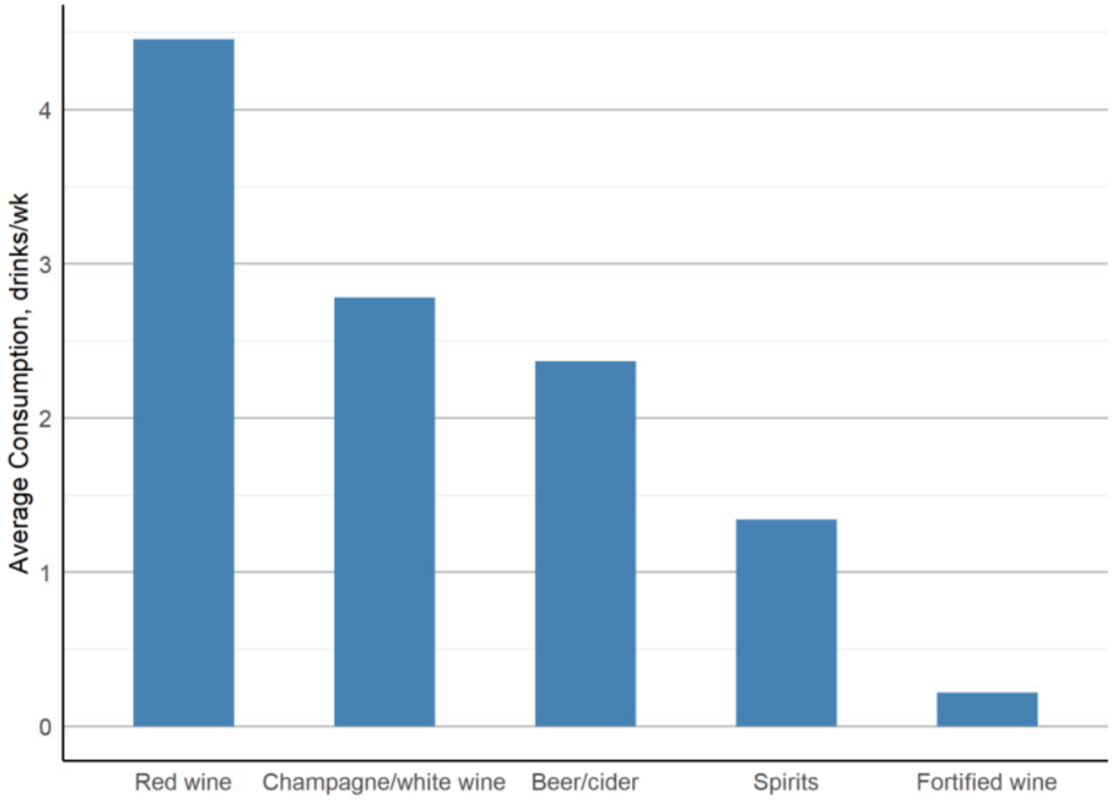

eFigure 5. Pure Ethanol Intake (g/day) From Specific Alcoholic Beverages and CSVD Burden Among Current Drinkers

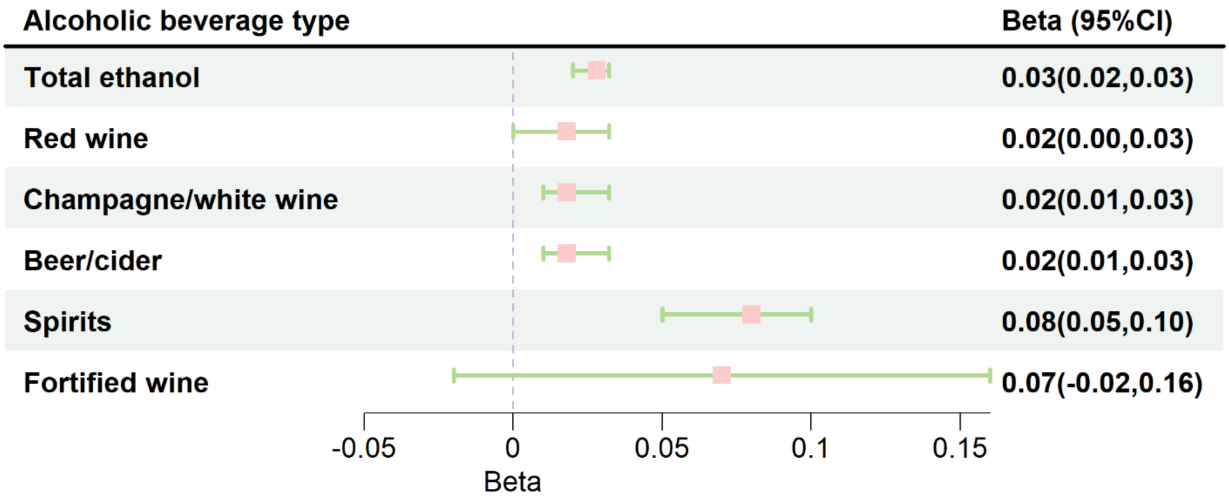

Abbreviations: CSVD, cerebral small vessel disease.

Result for analysis was adjusted for age, sex, ethnicity, TDI, education level, healthy physical activity, CVD family history, intake of fruits, intake of vegetables, eGFR, HDL-C, HbA1c, diabetes, hypertension and hyperlipidemia.
